# The National Association of College Faculties

**Published:** 1885-09

**Authors:** 


### Editorial, Etc.

---

THE NATIONAL ASSOCIATION OF COLLEGE FACULTIES.—The following is a synopsis of the proceedings of this Association at their late meeting held in Chicago. The Faculty of the University of Maryland, believing that dental students should have all the advantages offered by dental schools during the entire two-session course, which the graded course adopted by this Association does not afford, as it restricts students to one session only in operative dentistry, has declined to join the organization:

*Resolved*, That applicants for admission to our senior classes from foreign countries shall be required to furnish, properly attested, evidence of study, of attendance on lectures, etc., the same as is required of the junior students, and to pass the intermediate examinations.

*Resolved*, That a preliminary examination be required for entrance to our dental colleges; such requirements shall include a good English education. In case of any applicant failing to pass a satisfactory preliminary examination, the other colleges of this Association may be informed of the fact.

*Resolved*, That a candidate for matriculation who presents a diploma from a reputable literary institution, or other evidence of literary qualification, shall be admitted without further examination.

*Resolved*, That for preliminary examinations we recommend the following questions:

1. Write your name in full.
2. Give date of birth? Day, Month, Year.

3. Where were you born? Town, County, State.

4. Where do you now reside? Town, County, State.

5. What educational advantages have you had? Name the schools you have attended, and the time spent in each?

6. What branches have you studied, and to what extent have you pursued them?

7. In what occupation have you been engaged other than that of student, and how long were you thus employed?

8. When did you commence studying dentistry? Month, year.

9. How many months of actual medical and dental study have you had to date?

10. Have you attended a full course at any medical or dental school? If so, where and when?

11. With what preceptor, or preceptors, have you studied? Give name and present residence.

Sign your name in full.

Write an English composition, of at least two hundred words, upon a subject of the examiner's selection.

Further examination to be left to the judgment of the different faculties. The examination proposed is to embrace the following:

(1.) English Grammar. (2.) Arithmetic. (3.) Geography. (4.) Modern History. (5.) Government Topics.

A failure to pass the examinations may be communicated to other colleges.

*Resolved*, That the Colleges of this Association will receive into the senior class only such juniors as hold certificates of having passed a satisfactory examination in the studies of the junior year, this certificate to be a pledge to any college to which they may apply that a previous term has been properly spent in the institution whence they came.

*Resolved*, That the certificate shall read as follows:

This is to certify that..........................has attended one full course at the..........................College of Dental Surgery.......................... He was absent from lectures ...........weeks. He was absent from practical work........... weeks. He was examined at the close of the session, and found qualified to enter the senior class.

*Resolved*, That each student upon completing one regular course in any college represented in this Association shall be furnished with the above certificate, without presentation of which he shall not be accepted by any of our colleges for admission to the senior class.

Dr. Wilson then offered the following:

*Resolved*, That a committee of three be appointed to take into consideration the advisability of having a uniform practice in the furnishing of equipments for clinical work. Carried.

